# Automated functional upper limb evaluation of patients with Friedreich ataxia using serious games rehabilitation exercises

**DOI:** 10.1186/s12984-018-0430-7

**Published:** 2018-10-04

**Authors:** Bruno Bonnechère, Bart Jansen, Inès Haack, Lubos Omelina, Véronique Feipel, Serge Van Sint Jan, Massimo Pandolfo

**Affiliations:** 1 Laboratory of Anatomy, Biomechanics and Organogenesis (LABO) [CP 619], Université Libre de Bruxelles, Lennik Street 808, 1070 Brussels, Belgium; 2 Department of Electronics and Informatics – ETRO, Vrije Universiteit Brussel, Brussels, Belgium; 3 imec, Leuven, Belgium; 4 Laboratory of Functional Anatomy (LAF), Université Libre de Bruxelles, Brussels, Belgium; 5 Department of Neurology, Erasme Hospital, Brussels, Belgium

**Keywords:** Serious games, Assessment, Evaluation, Friedreich Ataxia, Kinect sensor

## Abstract

**Background:**

Friedreich ataxia (FRDA) is a disease with neurological and systemic involvement. Clinical assessment tools commonly used for FRDA become less effective in evaluating decay in patients with advanced FRDA, particularly when they are in a wheelchair. Further motor worsening mainly impairs upper limb function. In this study, we tested if serious games (SG) developed for rehabilitation can be used as an assessment tool for upper limb function even in patients with advanced FRDA.

**Methods:**

A specific SG has been developed for physical rehabilitation of patients suffering from neurologic diseases. The use of this SG, coupled with Kinect sensor, has been validated to perform functional evaluation of the upper limbs with healthy subjects across lifespan. Twenty-seven FRDA patients were included in the study. Patients were invited to perform upper limb rehabilitation exercises embedded in SG. Motions were recorded by the Kinect and clinically relevant parameters were extracted from the collected motions. We tested if the existence of correlations between the scores from the serious games and the severity of the disease using clinical assessment tools commonly used for FRDA. Results of patients were compared with a group a healthy subjects of similar age.

**Results:**

Very highly significant differences were found for time required to perform the exercise (increase of 76%, t(68) = 7.22, *P* < 0.001) and for accuracy (decrease of 6%, t(68) = − 3.69, *P* < 0.001) between patients and healthy subjects. Concerning the patients significant correlations were found between age and time (*R* = 0.65, *p* = 0.015), accuracy (*R* = − 0.75, *p* = 0.004) and the total displacement of upper limbs. (*R* = 0.55, *p* = 0.031). Statistically significant correlations were found between the age of diagnosis and speed related parameters.

**Conclusions:**

The results of this study indicate that SG reliably captures motor impairment of FRDA patients due to cerebellar and pyramidal involvement. Results also show that functional evaluation of FRDA patients can be performed during rehabilitation therapy embedded in games with the patient seated in a wheelchair.

**Trial registration:**

The study was approved as a component of the EFACTS study (Clinicaltrials.gov identifier NCT02069509, registered May 2010) by the local institutional Ethics Committee (ref. P2010/132).

## Background

Friedreich ataxia (FRDA) is an autosomal recessive disease with neurological and systemic involvement. Most commonly, loss of balance in a child or adolescent is the first symptom. Within a few years, trunk and limb ataxia become prominent, accompanied by dysarthria, oculomotor abnormalities and weakness. At the onset of disease, ataxia is mostly due to loss of large proprioceptive primary sensory neurons in the dorsal root ganglia (DRGs), with associated atrophy of the posterior columns and spinocerebellar tracts. With progression, cerebellar ataxia, due to atrophy of the dentate nucleus, becomes prevalent. Also with progression, distal axonal loss in the pyramidal tracts that cause further motor impairment [1, 2]. Most affected individuals become unable to walk within 10–15 years since disease onset, but then continue to deteriorate because of worsening upper limb ataxia, weakness, dysarthria, and eventually dysphagia. Clinical assessment tools commonly used for FRDA, such as the Scale for Assessment and Rating of Ataxia (SARA) [3], the Friedreich Ataxia Rating Scale (FARS) [4], and the International Cooperative Ataxia Rating Scale (ICARS) [5], become less effective in evaluating progression in patients with advanced FRDA, particularly when they are in a wheelchair and when upper limb function is impaired. This is partly due to a ceiling effect in these scales, in which gait and balance item have a major weight, but also to the characteristics of the disease [6, 7]. In FRDA, pyramidal symptoms like slowing and impairment of rapid alternating movements and difficulties in raising arms to point a target, eventually greatly affect performance in upper limb coordination tests, making their scoring more erratic. Hence the need for a more integrated tool to assess upper limb motor function in FRDA, particularly in wheelchair-bound patients with advanced disease.

Commercial video games have significantly evolved over the last decade. Today computer performance and play experience allow new perspectives for rehabilitation. Thanks to new gaming controllers (Nintendo Wii FitTM, Microsoft Xbox KinectTM, etc.) video game playing has changed from a passive (e.g., the player is seated on a sofa using a very simple controller) to an active experience: players have to move in order to interact with games (e.g., the player is truly active with a game controller requesting full body movements). Clinicians are now prospecting the new potential use of these games in rehabilitation mainly through testing available commercial games with patients suffering from various neurological pathologies (e.g. Cerebral Palsy [8], stroke [9], Parkinson disease [10]). Although encouraging results have been observed, especially in terms of motivation, there are several problems related to the use of commercial video games in rehabilitation. The player motion accuracy requested from the player during the games is low while most therapists will aim to improve patient joint control and coordination and the motion that must be performed to control the games do not correspond to rehabilitation exercises. Furthermore, there is currently no possibility to record the motion performed by the patients during those kind of exercises. However, collecting this information could be important to: (i) allow to provide direct feedback to patient and eventually correct the motion if they are not performed in the right way and (ii) to provide information to therapists in case of telerehabilitation exercises when the patient is performing exercises at home without the clinicians’ supervision [11].

In order to tackle the above-mentioned limitations, specific games must be developed for rehabilitation purposes [12]. Such kind of games, called serious games (SG) (i.e. games designed with a primary purpose other than pure entertainment), must be designed taking into account real clinical needs and constraints (e.g. simple visual background, based on relevant clinical schemes, range of motion and speed required to perform the exercises must be adaptable, etc.) [13] and allow to record motions performed by the patients [14, 15].

Depending on the joints and the kind of motions performed, results obtained with the Kinect sensor must be analyzed carefully. However, it was shown that using the displacement of wrist relative to trunk appears to be a good approach to evaluate upper limb function during rehabilitation exercises using serious games [15]. Furthermore, results obtained from Kinect and gold-standard optoelectronic device to assess upper limb functions are significantly correlated [16].

In this study, we tested if SG can be used as an assessment tool for upper limb function in patients with advanced FRDA. First, we determined if the scores obtained in the SG are able to differentiate patients and healthy participants. Then, we correlated SG results with genetic and clinical parameters of disease severity. To the authors best knowledge, there is currently no study about the use of SG to perform functional evaluation of patients with FRDA simultaneously with physical rehabilitation exercises.

## Methods

### Patients

Twenty-seven patients were included in the study, characteristics of the patients are presented in Table 1. They were all enrolled in the European Friedreich Ataxia Consortium for Translational Studies (EFACTS, Clinicaltrials.gov identifier NCT02069509) natural history study, the first prospective pan-European FRDA registry designed to define clinical rating scales and quality of life measures, which can be used in clinical trials. Since its inception in 2010, EFACTS has enrolled more than 800 genetically confirmed FRDA patients in nine European countries. SG was added to the EFACTS follow-up protocol at the Brussels site as an additional functional assessment. Data presented here are cross-sectional, for each patient they have been obtained at time of a single annual EFACTS visit. The study was approved by the local institutional Ethics Committee (ref. P2010/132) and patients gave written informed consent.

A database of healthy subjects ranging from 5 to 90 years was created in a previous study with the aim of using these data for comparison with patients by selecting the appropriate age range [15]. Exclusion criteria for the healthy subjects were neurological conditions, balance deficits or orthopedic disorders in the last six months. Forty-three healthy subjects have been used as an age-matched control group (26 (11) years old, 20 women).

**Table 1:** Characteristics of the patients included in the study

Variables	N	Mean (std)
Age (years)	27	26,0 (12.2)
Duration of the disease (years)	27	15.0 (7,44)
SARA score	27	22,5 (9,2)
ADL score	27	16,9 (6.7)
GAA-1 number of repeats	15	608.2 (306.4)
9 R 1 (s)	25	72.01 (42.3)
9 R 2 (s)	25	68.1(41.6)
9 L 1 (s)	25	87.2 (57.1)
9 L 2 (s)	25	79.3 (47.0)
CCFS F score (with writing)	25	1.33 (0.37)
CCFS H score (without writing)	25	1.31 (0.37)

### Clinical evaluation

The clinical assessment of EFACTS patients includes structured interviews, questionnaires, performance-based coordination tests, clinical disease rating scales and neuropsychological functional measures. Sampling of biomaterials and genetic analysis complement the examination. GAA repeat lengths for both alleles (GAA-1 and GAA-2) are determined at the ULB Laboratory of Experimental Neurology [17] Table 1.

Details of the clinical assessment have been previously reported [6]. Briefly, clinical tests whose scores have been correlated with those obtained from the SG include the Scale for the Assessment and Rating of Ataxia (SARA) [3], which quantifies ataxia symptoms based on eight items with a maximum score of 40; the Inventory of Non-Ataxia Symptoms (INAS) [18], which provides a checklist of non-ataxia symptoms such as changes in reflexes, other motor and sensory symptoms, ophthalmological findings, urinary dysfunction, and cognitive impairment; the Spinocerebellar Ataxia Functional Index (SCAFI), consisting of timed performance measures including an 8 m walk at maximum speed, the nine-hole peg test, and the rate of repeating the syllables “PATA” within 10s; the Cerebellar Composite Functional Score (CCFS) [19], a computerized set of timed tests of upper limb coordination performed with the dominant hand that includes a rapidly alternating click test, the 9-hole peg test and an optional writing test, which generates a composite score (higher values correspond to more impairment) including (F) or excluding (H) the optional writing test; the functional staging for ataxia part of the Friedreich Ataxia Rating Scale (FARS) [4]; the basic activities of daily living (ADL) part of the FARS was used to assess impairment in the ability to perform daily life activities.

### The serious game

**Fig. 1:**
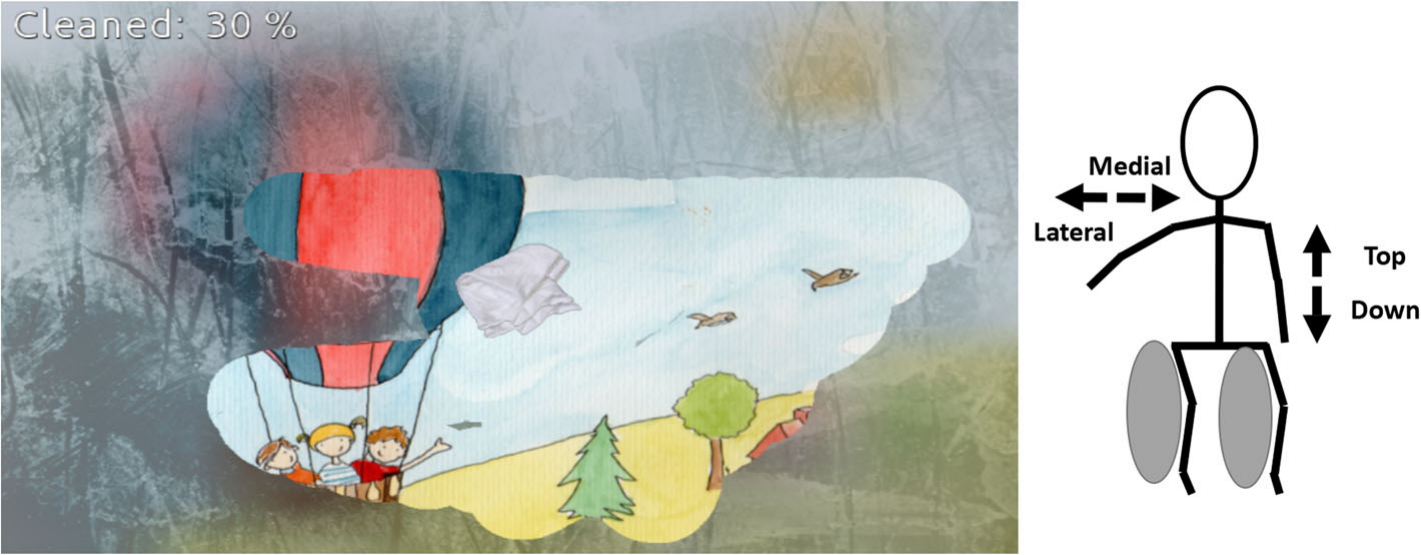
Screenshot of the game

Participants played one mini-game specially developed for physical rehabilitation as part of a previous project (called ICT4Rehab), the Wipe Out game [13]. The player has to clean the screen virtually covered by some virtual fog using a tissue controlled by medio-lateral and inferior-superior displacements of the upper limb (wrist) relative to the trunk (Fig. 1). Spatial displacements of the players were recorded by a Kinect sensor [14]. Motion data were stored in standard format (i.e., C3D) for further analysis. Participants were invited to stand in front of the screen and the sensor. Patients were asked to play the games three times. The mean of the three repetitions was used for statistical analysis. This was similar to previously published functional evaluation protocols using SG [15].

### Data processing

The use of this SG has been validated to perform functional evaluation of the upper limbs with healthy subjects across lifespan [15]. Two parameters were processed from the games: the time required to clean 90% of the screen and the accuracy. The accuracy of the motion was assessed by computing the number of times that the subject is placing the cloth in the same position on the screen (Eq.1). The number of frames where the cloth was in a position that had already been cleaned were computed (“cleaned” frames). The result was finally expressed in percentage. The higher the accuracy score, the better the results in term of performance.

1 $$ \mathrm{accuracy}=100-\left(\left(\frac{{\mathrm{Number}\ \mathrm{of}}^{"}{\mathrm{cleaned}}^{"}\ \mathrm{frames}}{\mathrm{Total}\ \mathrm{number}\ \mathrm{of}\ \mathrm{frames}\ }\right)\times 100\right) $$

Several parameters were then processed from the motion performed by the patients and recorded by the Kinect, those parameters are adapted from previous studies on the validation of scores to assess dynamic balance during rehabilitation exercises [20]: the total displacement of the wrist related to the trunk (DOT) based on medio-lateral (ML) and top-down (TD) displacements (Eq. 2), the area of 95% confidence prediction ellipse (Area) (Eq. 3), the dispersion of the trajectory from the mean position in ML and TD directions (RMS_ML_ and RMS_TD_) (Eqs. 4 and 5), the range of displacement (R_ML_ and R_TD_ (Eqs. 6 and 7), the mean velocity displacement (MV_ML_ and MV_TD_) (Eqs. 8 and 9) and the total mean velocity (TMV) (Eq. 10).

2 $$ DOT={\sum}_{i=1}^N\sqrt{ML{(i)}^2+ TD{(i)}^2} $$

3 $$ Area=\pi \times prod\left(2.4478\times \sqrt{svd\left( eig\left(\mathit{\operatorname{cov}}\left( ML, TD\right)\right)\right)}\right) $$

4 $$ {RMS}_{ML}=\frac{1}{N}\sqrt{\sum_{i=1}^N ML{(i)}^2} $$

5 $$ {RMS}_{TD}=\frac{1}{N}\sqrt{\sum_{i=1}^N TD{(i)}^2} $$

6 $$ {R}_{ML}=\max (ML)-\min (ML) $$

7 $$ {R}_{TD}=\max (TD)-\min (TD) $$

8 $$ {MV}_{ML}=\frac{f}{N}\ {\sum}_{i=1}^{N-1}\left| ML\left(i+1\right)- ML(i)\right| $$

9 $$ {MV}_{TD}=\frac{f}{N}\ {\sum}_{i=1}^{N-1}\left| TD\left(i+1\right)- TD(i)\right| $$

10 $$ TMV=\frac{f}{N}\ {\sum}_{i=1}^{N-1}\sqrt{{\left( ML\left(i+1\right)- ML(i)\right)}^2+{\left( TD\left(i+1\right)- TD(i)\right)}^2} $$

Data were processed and plotted using a customized software routine developed in Matlab 2017.

### Statistics

To detect a difference of 40% of the time required to perform the exercise between patients and control with 80% power and a two-sided type I error of 5%, we calculated that we need to include 24 patients.

Normality of each parameter was checked using the Shapiro-Wilk tests and by graphical analysis (histogram, boxplot and normal Q-Q plot). Pearson’s correlation coefficients were used to determine if the scores from the SG were correlated with the clinical evaluation. Independent sample T-test were applied to compare patients and healthy subjects. Statistics were performed with SPSS 20 and RStudio (version 1.1.442) with R version 3.4.4, significance level was set at 0.05.

## Results

**Fig. 2:**
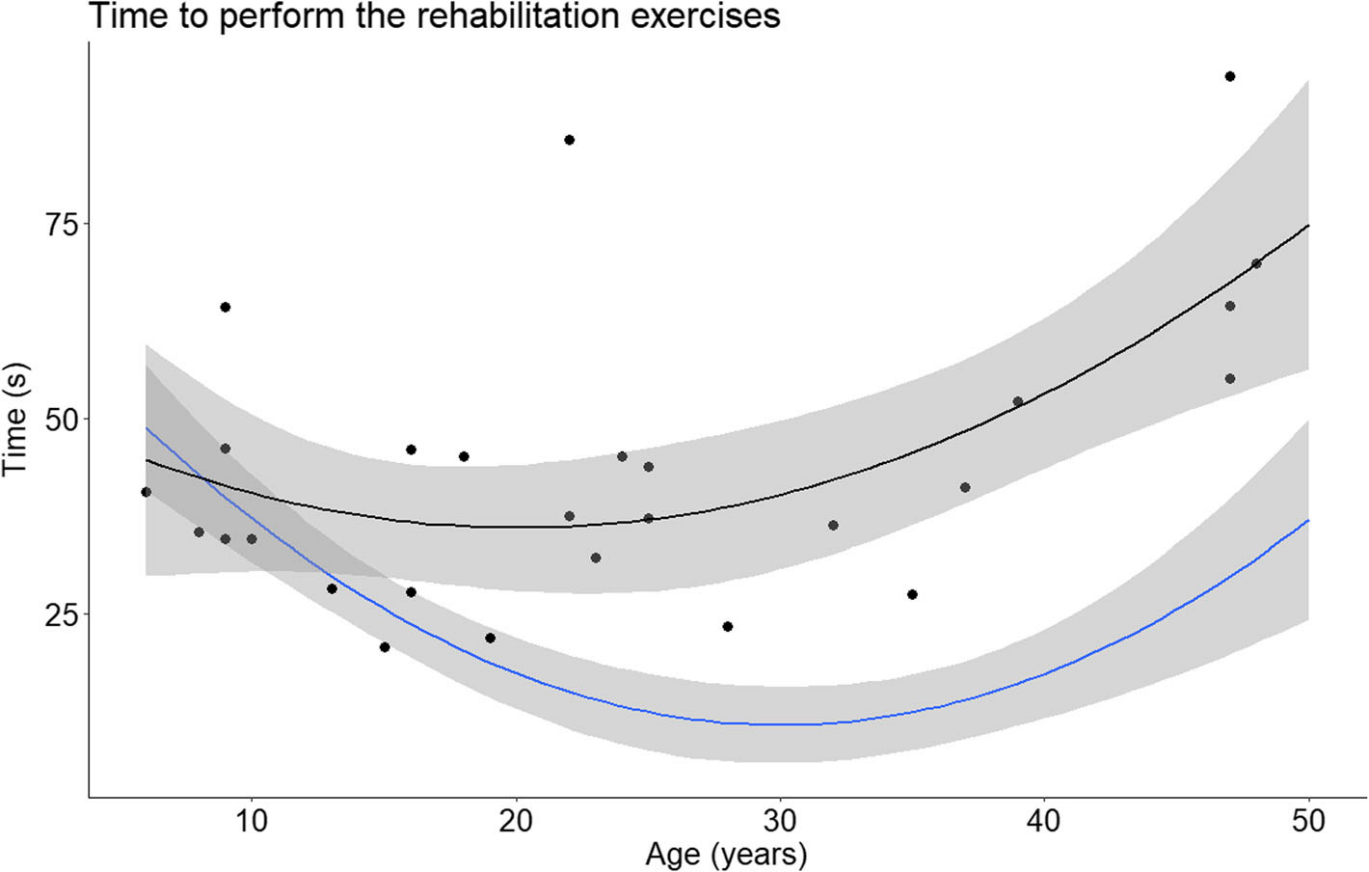
Mean and 95% confidence interval (CI) of the time for healthy subjects (blue) and patient results (black). Black dots represent individual results of the patients

**Fig. 3:**
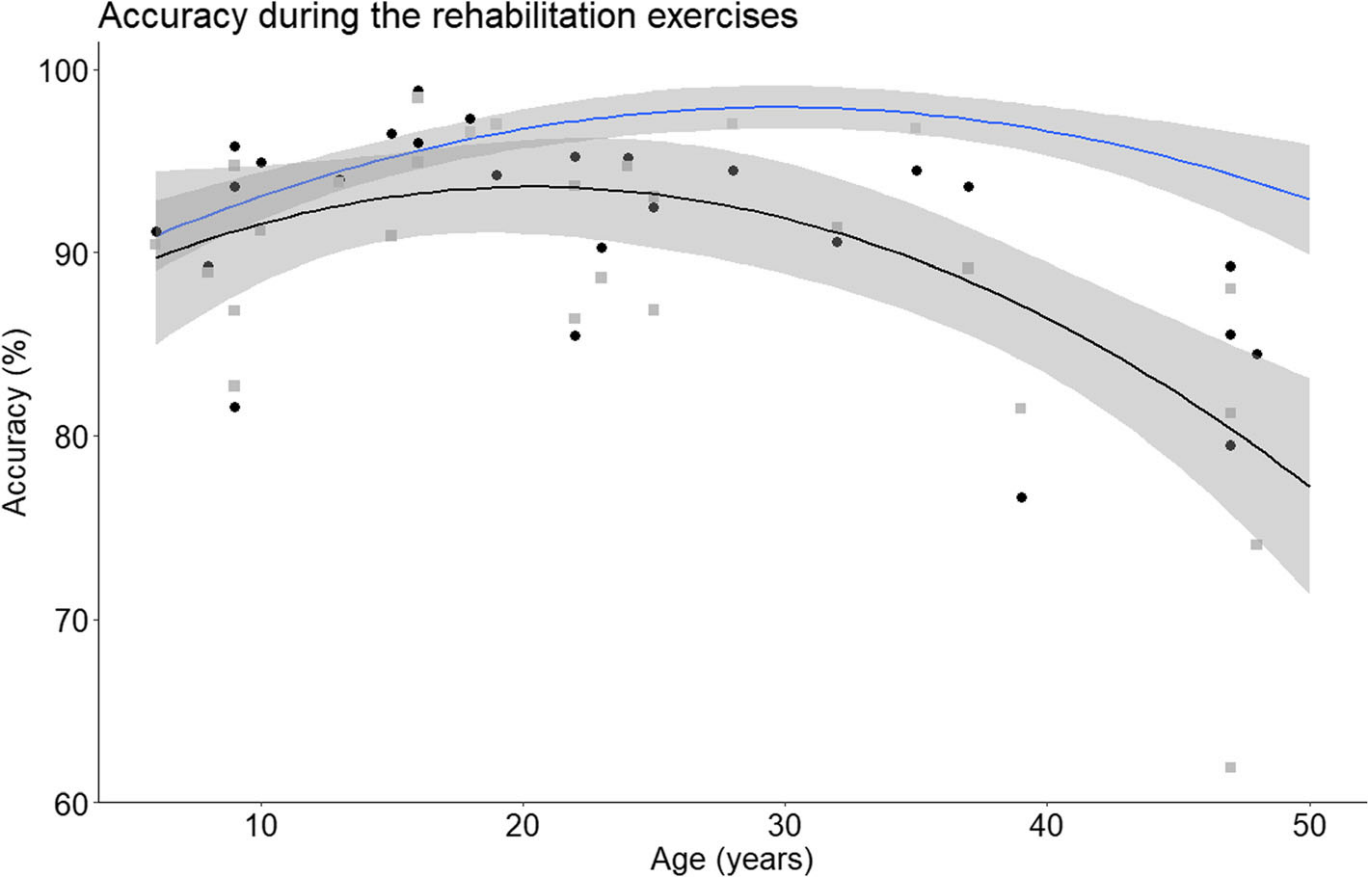
Mean and 95% CI of the accuracy for healthy subjects (blue) and patient results (black). Black dots (right side) and grey squares (left side) represent individual results of the patients. Since no statistically significant difference were found between right and left side mean of the two sides was used for the fitting

Age is an important factor for both gross and fine motor control [21, 22] and since the range of age is important in our group of patients (from 6 to 48 years old) results for time and accuracy were plotted according to the age of the participants and compared to results of the healthy control group in Figs. 2 and 3 respectively. Quadratic fitting with 95% CI were plotted since it has been previously demonstrated that this fitting was the most adapted for this kind of data [15]. Very highly significant differences were found for time (increased of 76%, t(68) = 7.22, *P* < 0.001) and for accuracy (decreased of 6%, t(68) = − 3.69, *P* < 0.001) between healthy subjects and patients. Results of all the studied parameters are presented in Table 2. Since no significant differences were found between right and left side for any of the parameters mean values were presented.

**Table 2:** Mean (std) results for patients and control

Variables	Patients (*n =* 27)	Control (*n =* 43)	T-test	Difference [95% CI]
Time (s)	44 (18)	25 (15)	t(68) = 7.22, *P* < 0.001	19 [12; 29]
Accuracy (%)	88 (7)	94 (6)	t(68) = 3.69, *P* < 0.001	6 [3; 9]
DOT (cm)	1462 (664)	1150 (350)	t(68) = 2.24, *P* = 0.026	312 [40; 583]
Area (cm^2^)	1421 (913)	1278 (576)	t(68) = 0.74, *P* = 0.458	143 [− 242; 528]
RMS_ML_ (cm)	10 (3)	8 (4)	t(68) = 2.38, *P* = 0.018	2 [0.3; 3.7]
RMS_TD_ (cm)	7 (3)	5 (2)	t(68) = 3.06, *P* = 0.003	2 [0.7; 3.3]
R_ML_ (cm)	55 (11)	48 (8)	t(68) = 2.86, *P* = 0.005	7 [2.2; 11.8]
R_TD_ (cm)	44 (10)	39 (7)	t(68) = 2.27, *P* = 0.024	5 [0.7; 9.3]
MV_ML_ (cm/s)	38 (16)	56 (10)	t(68) = 5.23, *P* < 0.001	18 [13; 24]
MV_TD_ (cm/s)	33 (14)	53 (9)	t(68) = 6.61, *P* < 0.001	20 [14; 26]
TMV (cm/s)	57 (20)	81 (10)	t(68) = 5.19, *P* < 0.001	26 [16; 32]

*P*-value is the results of T-tests

Pearson’s correlation coefficients are presented in Table 3. Statistically significant correlations were found between age and Time (*R* = 0.65, *p* = 0.015), accuracy (*R* = − 0.75, *p* = 0.004) and the total displacement of upper limbs (DOT) (*R* = 0.55, *p* = 0.031). Statistically significant correlations were found between the age of diagnosis and the speed related parameters (*R* = − 0.53, *p* = 0.021 and *R* = − 0.49, *p* = 0.029 for MV_ML_ and MV_IS_ respectively).

**Table 3:** Pearson’s correlation coefficients (R) between scores obtained from the SG and the clinical evaluation

Variables	Time	Accuracy	DOT	Area	RMS _ML_	RMS_TD_	R_ML_	R_TD_	MV _ML_	MV _TD_	TMV
Age (year)	.63**	−.73**	.57*	−.16	−0.04	−.20	−.06	−.20	−.16	−.20	−.41
Age of diagnosis (year)	.44	−.64**	.28	−.10	−.23	−.12	−.18	−.36	−.53*	−.49*	−.19
Duration of the disease (year)	.64**	−.67**	.48**	−.25	.26	.26	.32	.06	−.16	−.25	−.25
SARA score	.27	−.21	.46*	.32	.35	.24	.32	.29	.25	.21	.21
ADL score	.13	.15	**.38**	.37	.50*	.59*	.39*	.50*	.36	.28	.28
GAA-1 number of repeats	−.18	.28	.61*	.39	.47*	.58*	.64**	.39	.26	.01	.11
9 R 1 (s)	.14	.22	.61*	.64*	.01	.59*	.50*	.49*	.38*	.46*	.41*
9 R 2 (s)	.15	.0.20	.61*	.63*	−.02	.55*	.48*	.50*	.37	.46*	.41*
9 L 1 (s)	.18	.65**	.61*	.51*	.36	.59*	.41	.48*	.30	.40*	.32
9 L 2 (s)	−.16	0.11	.63*	.60*	.12	.54*	.43*	.49*	.32	.41*	.24
CCFS F score (with writing)	−.13	.29	.16	.36	−.13	.15	−.43*	.18	.47*	.37	.44*
CCFS H score (without writing)	−.18	.37	.11	.33	.23	.26	.31	.19	.30	.27	.30

*Significant correlation (*P* < .05)

**Significant correlation (*P* < .01)

Statistically significant correlations were found for TD displacements (*R* = 0.48 and 0.47 for ranges and RMS) and the basic activities of daily living.

Correlations were found between the nine-hole peg tests and the DOT (*R* = 0.60, *p* = 0.012), the area (*R* = 0.59, *p* = 0.025), the amplitudes (*R* = 0.45, *p* = 0.041 and *R* = 0.49, *p* = 0.033 for ML and TD displacements respectively). For the speed correlations were found only for the TD direction (*R* = 0.43, *p* = 0.030) not for ML.

Concerning the comparison with the genetic analysis statistically significant correlations were found between the parameters extracted from the SG (DOT, RMS_ML_, RMS_TD_ and R_ML_) and GAA-1.

## Discussion

During the course of FRDA, patients gradually become unable to stand and walk. Therefore, it is essential that an upper limb coordination test like SG can be performed with the patient seated in a wheelchair. Although previous studies reported some limitations of the Kinect sensor to measure upper limb joint angles of patients in wheelchairs [23], this study demonstrates it is feasible to combine rehabilitation exercises and functional evaluation of FRDA patients, even the more disabled ones.

The first aim of this study was to determine if functional analysis performed with the SG can be used to differentiate patient and healthy subjects. Statistically significant differences were obtained for most of the studied parameters except for the area (*p* = 0.37) indicating that the patients stay within an acceptable range in the limits of the screen. By separately analyzing the two components (medio-lateral and top-down) of motion we observed that the difference is due to a too large amplitude in the ML direction whereas there is no difference at the TD level.

**Fig. 4:**
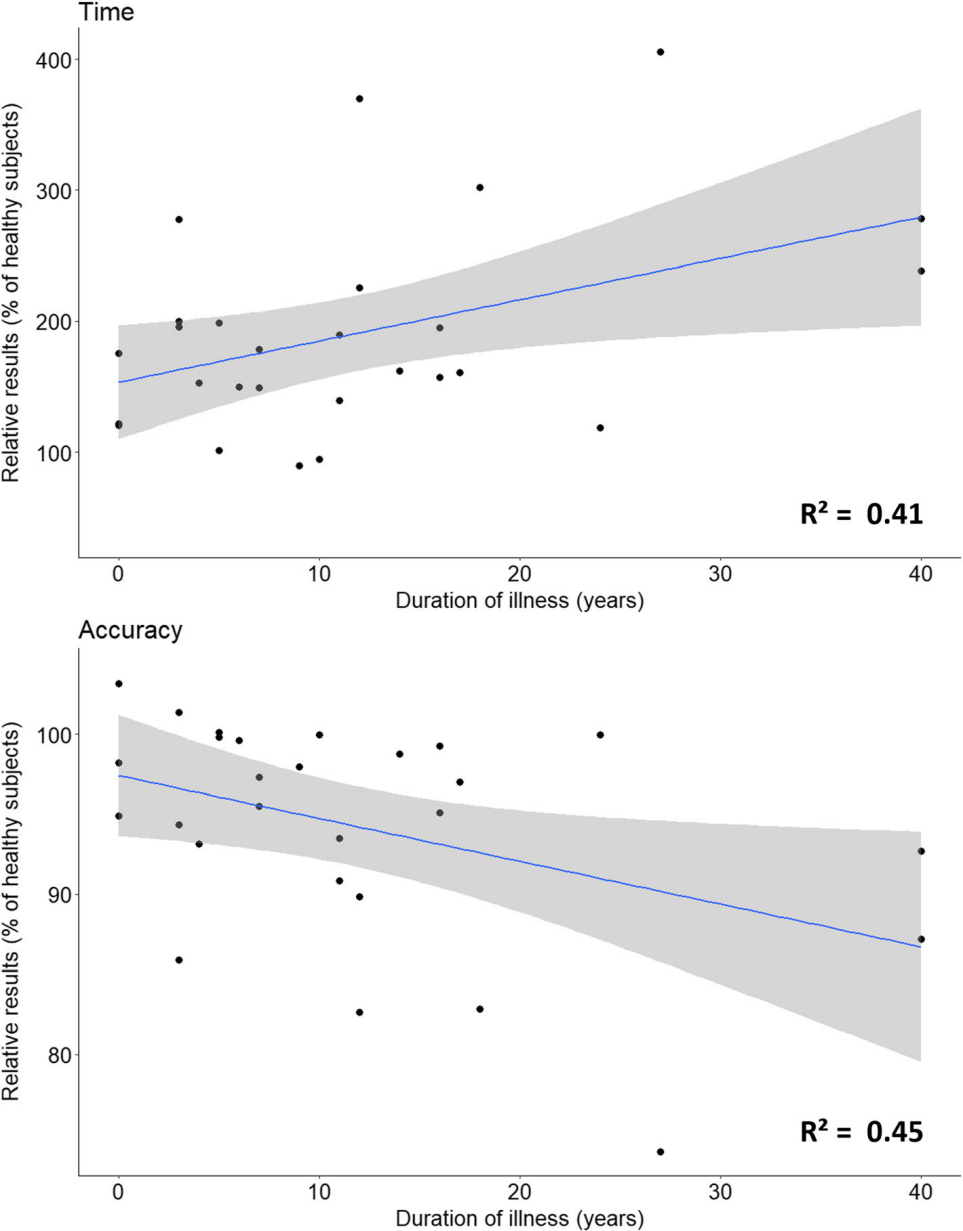
Results of the time and accuracy expressed in percentage of the values of healthy subjects according to the duration of the disease. Linear fitting with 95% CI is presented with R^2^

**Fig. 5:**
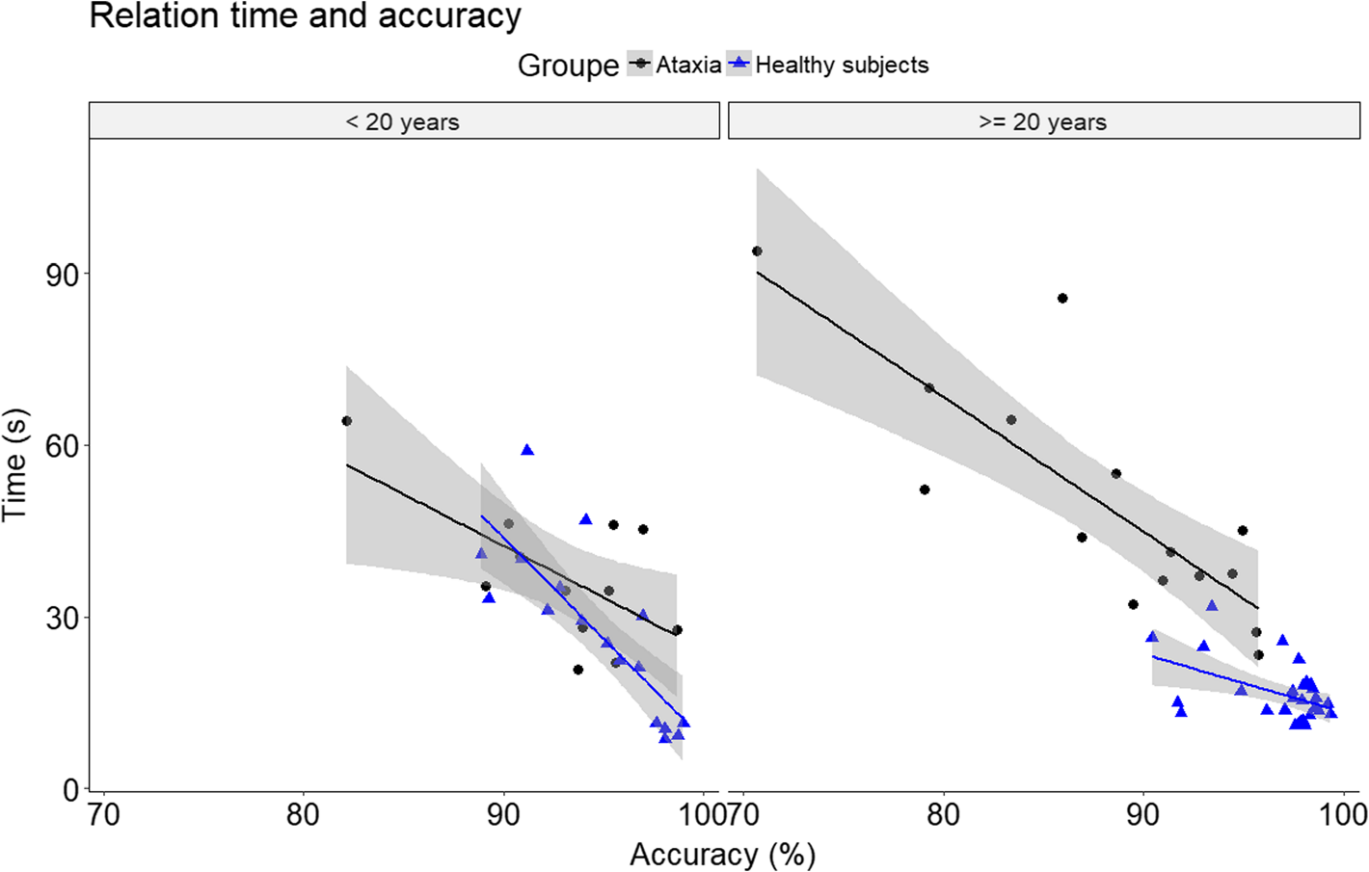
Scatter plots and linear regression for the relation between time and accuracy according to age

Figures 2 and 3 show that until the age of 20 patients seem to remain in the confidence interval of the control group, differences only appeared later in the course of the disease. Similar trends were found for all parameters. In order to better visualize this phenomenon, results were normalized and expressed as a percentage of control group values. In Fig. 4 results are plotted according to disease duration. There was a statistically significant correlation between disease duration and decreased speed (*R* = 0,64, *p* = 0,002). Interestingly, the decrease in speed was associated with a significant reduction of accuracy (*R* = − 0,67, *p* = 0,001). We also analyzed the relation between speed and accuracy. For FRDA there is a higher correlation (*R* = − 0.87, *p* < 0.001) than for healthy subjects (*R* = − 0.72, *p* < 0.001). We divided the subjects according to age (bellow or above 20 years old) and plotted the results in Fig. 5. For participants aged less than 20 years the slope of the regression line was β = − 1.8, *p* = 0.022, R^2^ = 0.42 for FRDA and β = − 3.5, *p* < 0.001, R^2^ = 0.54 for healthy subjects. For participants older than 20 years the slope of the regression line is: β = − 2.3, *p* < 0.001, R^2^ = 0.68 for FRDA and β = − 1.4, *p* < 0.001, R^2^ = 0.46 for healthy subjects.

These results indicate that SG reliably captures motor impairment of FRDA patients due to cerebellar and pyramidal involvement. Results also show that functional evaluation of FRDA patients can be performed during rehabilitation therapy embedded in games. Younger patients who still perform within the confidence interval of the control group are likely to have a mainly afferent ataxia due to DRG pathology, so that it is probable that their motor performance under visual control is not degraded. The fact that GAA-1 correlates with several SG parameters is in agreement with other observations that the severity of the genetic mutation not only affects the age of symptom onset, but also the subsequent rate of progression and severity of symptoms [7]. Analysis of a larger sample and prospective follow-up of patients will assess the value of SG as a potential outcome measure for clinical trials, particularly in more advanced patients for which current evaluation tools becomes less effective with the evolution of the disease.

One limitation of this study is that only one session of measurement has been done, because patients only come once a year to the hospital for the EFACTS follow-up. Therefore, longitudinal studies are needed to determine the reproducibility of measurements and the influence of training, habituation or fatigue on the results.

Finally, the last point is to integrate these solutions into patients’ home to motivate them to perform the rehabilitation exercises. In addition to this aspect of motivation these kinds of solutions allow and evaluation of the quality of the rehabilitation exercise and thus a longitudinal follow-up for both patients and clinicians.

## Conclusion

The use of new technologies in rehabilitation, including SG, is becoming increasingly important. In this study demonstrated that it is possible to combine rehabilitation exercises using SG and automated upper limb functional assessment in FRDA patients in wheelchairs. Future works are needed to determine if such kind of solution can be successfully integrated in the rehabilitation program and whether the kind of data presented in this paper can be used to predict disease progression.

## Abbreviations

ADL: Activity of daily living
DOT: Total displacement of the wrist related to the trunk
DRGs: Dorsal root ganglia
EFACTS: European Friedreich Ataxia Consortium for Translational Studies
FARS: Friedreich Ataxia Rating Scale
FRDA: Friedreich ataxia
ICARS: International Cooperative Ataxia Rating Scale
ML: Medio-lateral
MV: Mean velocity
R: Range
RMS: Dispersion of the trajectory from the mean position
SARA: Scale for Assessment and Rating of Ataxia
SG: Serious games
TD: Top-down
TMV: Total mean velocity
